# Guided diffusion for molecular generation with interaction prompt

**DOI:** 10.1093/bib/bbae174

**Published:** 2024-04-21

**Authors:** Peng Wu, Huabin Du, Yingchao Yan, Tzong-Yi Lee, Chen Bai, Song Wu

**Affiliations:** Department of Urology, South China Hospital, Medical School, Shenzhen University, Fuxin Road, Longgang District, Shenzhen, 518116, China. Tel.: +86 0755 89798999; MoMed Biotechnology Co., Ltd., Hangzhou 310005, China; MoMed Biotechnology Co., Ltd., Hangzhou 310005, China; Institute of Bioinformatics and Systems Biology, National Yang Ming Chiao Tung University, Hsinchu 300, Taiwan, China. Tel.:+886 0928 560313; MoMed Biotechnology Co., Ltd., Hangzhou 310005, China; Warshel Institute for Computational Biology, School of Life and Health Sciences, School of Medicine, The Chinese University of Hong Kong, Shenzhen, Shenzhen, 518172, Guangdong, China. Tel.:+86 0755 84273118; Department of Urology, South China Hospital, Medical School, Shenzhen University, Fuxin Road, Longgang District, Shenzhen, 518116, China. Tel.: +86 0755 89798999; South China Hospital, Health Science Center, Shenzhen University, Shenzhen 518116, China

**Keywords:** molecular generative model, prompt learning, diffusion model, atomic interaction

## Abstract

Molecular generative models have exhibited promising capabilities in designing molecules from scratch with high binding affinities in a predetermined protein pocket, offering potential synergies with traditional structural-based drug design strategy. However, the generative processes of such models are random and the atomic interaction information between ligand and protein are ignored. On the other hand, the ligand has high propensity to bind with residues called hotspots. Hotspot residues contribute to the majority of the binding free energies and have been recognized as appealing targets for designed molecules. In this work, we develop an interaction prompt guided diffusion model, InterDiff to deal with the challenges. Four kinds of atomic interactions are involved in our model and represented as learnable vector embeddings. These embeddings serve as conditions for individual residue to guide the molecular generative process. Comprehensive *in silico* experiments evince that our model could generate molecules with desired ligand–protein interactions in a guidable way. Furthermore, we validate InterDiff on two realistic protein-based therapeutic agents. Results show that InterDiff could generate molecules with better or similar binding mode compared to known targeted drugs.

## INTRODUCTION

Structure-based drug discovery (SBDD) aims to design molecules that can bind to a target protein with notable binding affinity and selectivity, which acts as a pivotal strategy in contemporary biopharmaceutical investigations [1]. However, SBDD encounters challenges due to the vast chemical space. It is estimated that the realm of ‘drug-like’ molecules range from 10^20^ to 10^60^, taking into account oral bioavailability and Lipinski’s rule of five [2]. Conventional computer-aided SBDD methods, such as virtual screening, are computationally costly due to the large feasible chemical space and the fact that we could not find novel drugs. In recent years, molecular generative models have emerged as a promising technique in drug discovery and enabled *de novo* molecular design. Unlike classification or regression models, which are commonly used for predicting or classifying the attributes of molecules, molecular generative models learn the transformation process from a specified distribution to the target data distribution, with the goal of generating novel data instances. Earlier methods relied on one-dimensional (1D; SMILES strings and SELFIES strings) or two-dimensional (graphs) molecular representations [3–6], which successfully generated diverse and novel molecules. Nevertheless, these models overlooked three-dimensional (3D) spatial information of molecules and the protein pocket environment, which are essential for molecular properties and protein binding affinity. Consequently, 3D structure–based generative methods have gained significant attention due to their capability of designing molecules that bind to a specific protein pocket.

Various models have recently been proposed for 3D structure–based molecular generation, encompassing variational autoencoders (VAEs), flow-based models, autoregressive models and diffusion models [7–14]. In their work [7], Matthew *et al.* represent molecules through density grids and employ a conditional VAE is used to generate new atomic density grids. Atom fitting and bond inference are applied to obtain novel molecules. While they achieve remarkable results in generating diverse molecules, as pointed in Schneuing *et al*., Guan *et al*. and Huang [9, 10, 13], inferencing molecules from density grids is a non-trivial task and irregularities are presented in the generated molecules. Moreover, the model is not equivariant and hard to scale to large protein systems. Peng *et al.* utilize an autoregressive model to generate molecules atom by atom within protein pocket [14]. However, this generation process is inefficient and prone to cumulative deviations due to the sequential nature of generation. For instance, if the first several atoms are placed at improper positions, subsequent generations will be biased. In contrast, diffusion-based models sample atom types and coordinates simultaneously, considering the protein context. Specifically, diffusion model defines a noise schedule and add noise to the molecular geometry in the forward process. In the backward (generation) process, the model learns to reverse the noise process to recover the true molecular geometry. There is no mismatch between the training and generating process in diffusion models.

Nonetheless, diffusion models still face one limitation in real scenarios. Essentially, diffusion models pertain to a model class named score-based generative models [15]. Another member in score-based generative models is score matching, which estimates the score of data at different noise scales and samples by gradually decreasing noise levels [16, 17]. As Song *et al.* pointed, when the number of noise scales go to infinity, score-based generative models can be regarded as a stochastic differential equation (SDE) [15]. While sampling from the SDE, an associated ordinary differential equation (ODE) exists that shares the same marginal probability densities. However, this correspondence leads to a decrease in the diversity of generated molecules [12]. Additionally, the binding mode of proteins with ligand is vital for elucidating the biological processes. It has been found that only a fraction of residues in the pocket, called hotspots, contribute most to the binding affinity [18, 19]. A mutation in hotspots can cause significant drop in binding affinity and even induce drug resistance in patients [20, 21]. In contemporary development of drugs, hotspots are crucial for rational drug design, and one usually desires that the drug can form interactions with hotspots. However, current diffusion models overlook the protein–ligand interaction information and cannot customize the generated molecules.

Inspired by the fruitful progress of prompt-based learning in nature language processing, we develop a prompt-based diffusion model called InterDiff to tailor the binding mode of generated molecules in the protein pocket. Our study primarily makes contributions in three aspects. Firstly, we introduce a novel approach by explicitly integrating interaction information into the diffusion generative model to guide molecular design.. Secondly, to enable the model to capture long-range interactions between ligands and proteins, we design a cross-attention module. This module computes attention between ligand and protein atoms, incorporating both interaction and distance information. Lastly, we conduct experiments on real targets, demonstrating the applicability of our method to real-world targets and the ability to reproduce critical interactions between drugs and targets. Specifically, we introduce four kinds of learnable prompt embedding to indicate the interaction type of protein residues, including π-π interaction, cation-π interaction, hydrogen bond interaction and halogen bond interaction. In addition, we used one-hot encoded discrete interaction prompts to distinguish protein residues involved in different interactions. We perform an empirical study on the CrossDocked2020 dataset and show that InterDiff is able to generate molecules under prescribed interaction prompts with high probability. In addition, we validate our model on two well-known targets in neural systems and cancers, respectively. To our knowledge, this is the first study to incorporate interaction prompts in structure-based drug design.

## RESULTS

**Figure 1:**
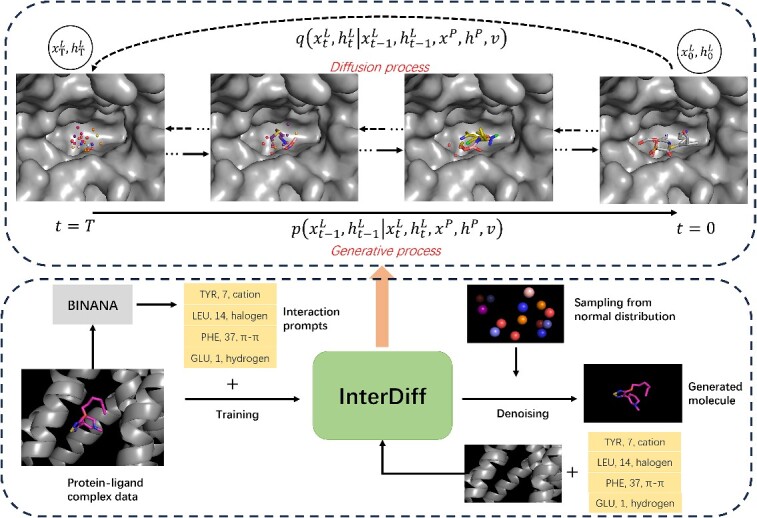
Workflow of InterDiff in a protein conditional generation. In diffusion process $q$, we simulate a progressively noised ligand point cloud (ligand coordinates and atom types: ${x}^{(L)},{h}^{(L)}$, $(L)$ indicates ligand) under protein environment (protein coordinates and atom types: ${x}^{(P)},{h}^{(P)}$, $(P)$ indicates protein) over $T$ timesteps ($T=1000$ in the experiment). The interaction prompts $v$ indicates the interaction types for each residue (‘*TYR, 7, cation’*: the 7th residue is tyrosine with cation-π interaction), which are extracted by BINANA and are tunable in the diffusion process. In the generative process $p$, a neural network learns to recover data from a noise distribution conditioned on protein and prompts.

InterDiff leverages interaction prompts to guide molecular generative model and design molecules, resembling the prefix-tuning that optimizes a small continuous task-specific vector (Figure 1 and methods part) [22]. As shown in Figure 1, a diffusion process is learned to transform the ligand data distribution into a normal distribution conditional on the protein environment and interactions. In the generative process, the protein pocket and interactions serve as the conditions to guide each denoising step and design molecules that satisfy certain interactions. To evaluate InterDiff, we first conduct the experiment on two benchmark datasets compared with five recently published methods on eight general metrics. The second dataset is utilized for external validation. Furthermore, we evaluate the model performance in generating molecules with predetermined interactions, a pivotal feature of InterDiff. We found that InterDiff can design molecules with specified interactions with high accuracy. Additionally, we tested on real targets and discovered that the model can generate molecules with interaction patterns akin to existing drugs, as well as design new molecular fragments on the molecular scaffold.

### Data

The InterDiff model was trained and evaluated using the crossDocked2020 dataset [23]. This dataset was derived from the Protein Data Bank (PDB) database. We follow the same splitting and filtering criterion described in Guan *et al*. and Peng *et al*. [10, 14], obtaining 100 000 samples for training and 100 samples for testing (data points whose binding pose RMSD is greater than 1 Å are filtered). The protein–ligand interactions are detected by BINANA2 [24], encompassing four distinct interactions including cation-π, π-π, hydrogen and halogen interaction (Table S3). We exclude the complexes that have residue detected with more than one interaction. Phenylalanine and tyrosine were observed to have four interactions (Figure S1) and a hydrogen bond account for the vast majority of interactions (Figures S1 and S2). For external validation, we utilized the DOCKSTRING dataset [25]. DOCKSTRING has docking poses of more than 260 000 molecules for each protein and 58 clinically relevant targets for test.

#### Molecular structure and properties

Routinely, we assess the generated molecules from test set in eight metrics: (1) Vina Score: A binding affinity indicator calculated by physical-based empirical scoring function. (2) QED: QED is a measurement of drug-likeness of molecules. (3) SA: SA (Synthetic Accessibility) measures the feasibility of synthesized molecules based on fragmental analysis in a compound database. (4) Lipinski: we calculate the Lipinski score by quantifying how many rules are fulfilled in Lipinski’s rule of five. (5) Diversity: diversity is computed by averaging the pairwise dissimilarity (one minus *Tanimoto* similarity) of generated molecules in each pocket. (6) Validity: validity measures the proportion of generated molecules that pass RDKit valency check. (7) Novelty: the proportion of molecules that do not present in test set SMILES. Since there are more than 260 000 molecules for each target in the DOCKSTRING test dataset, we do not report the Novelty metric for this dataset. (8) Uniqueness: the proportion of non-repetitive molecules in all generated molecules.

**Figure 2:**
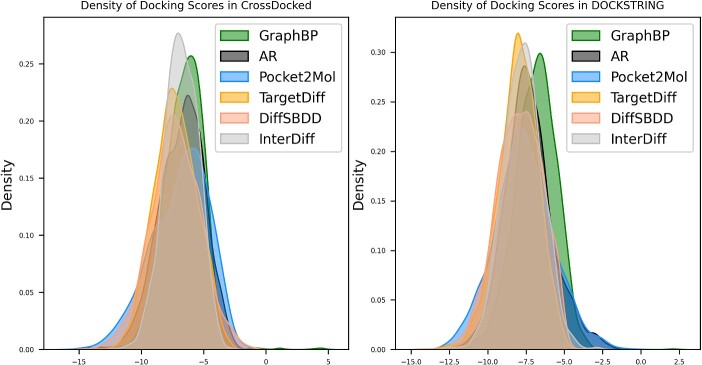
Density plot of docking scores for InterDiff and baseline methods.

Table 1 displays the results of InterDiff and baseline methods. Overall, InterDiff performs well in diversity and the other indicators are less ideal. Compared to the reference molecules in the test set, InterDiff has similar Lipinski scores, but lower QED and SA scores. However, this also indicates that InterDiff could generate novel molecules since QED and SA are calculated based on the existing drug database. TargetDiff and DiffSBDD achieve best results in Vina score, and Pocket2Mol performs best in QED, SA and Lipinski. It is worth noting that Pocket2mol performs better than the molecules in the test set in terms of QED, SA and Lipinski. This may be attributed to its use of the geometric vector perception module [26, 27], which better preserves molecular geometric features and explicitly incorporates chemical bonds during the generation process. Pocket2mol explicitly specifies chemical bond information during the training and sampling process, which explains its high performance in metrics such as QED, SA and Lipinski. To evaluate the substructure characteristics of generated molecules, we analyze the distribution of different ring sizes. Results show that diffusion-based models tend to produce a larger proportion of seven-membered rings while GraphBP has more three-membered rings. (Table S1). We speculate that the diffusion model tends to generate multi-atomic rings due to its lack of explicit modeling of chemical bonds. During the process of molecular reconstruction, when an atom is in close proximity to an already identified six-membered ring, RDkit will incorporate it into the six-membered ring, turning it into a seven-membered ring, without considering the overall structure. Besides, we observed that the variance of the Vina score in InterDiff is significantly smaller compared to other methods, as depicted in Figure 2 and Table 1. During the generative process, we maintain the consistency in the interaction prompt of the protein residues, as described in the Methods section. These prompts are aligned with the reference molecules in the test set, and the number of atoms is sampled according to the joint distribution of pocket size and number of atoms in ligand (Figure S3). The interaction prompt for the residues restricts the fluctuating extent of binding pose of the generated molecules, resulting in a binding mode akin to the reference molecules.

**Table 1:** Evaluation results from test set of CrossDocked2020 dataset. The performance is re-evaluated for baseline methods GraphBP, Pocket2Mol, 3D-SBDD, TargetDiff and DiffSBDD. The autoregression based methods (GraphBP, Pocket2Mol, 3D-SBDD) discard the repeated molecules in the generative process, so we do not report the Novelty and Uniqueness for these three methods

		Vina score ($\downarrow$)	QED ($\uparrow$)	SA ($\uparrow$)	Lipinski ($\uparrow$)	Diversity ($\uparrow$)	Validity ($\uparrow$)	Novelty ($\uparrow$)	Uniqueness ($\uparrow$)
CrossDocked 2020	Test set	−6.871$\pm$2.32	0.476$\pm$0.20	0.728$\pm$0.14	4.340$\pm$1.14	$-$	$-$	$-$	$-$
	GraphBP	−6.54$\pm$2.40	0.501$\pm$0.14	0.417$\pm$0.09	4.872$\pm$0.41	0.815$\pm$0.01	99.68%	$-$	$-$
	Pocket2Mol	−6.561$\pm$2.67	0.573$\pm$0.16	0.756$\pm$0.13	4.879$\pm$0.42	0.731$\pm$0.12	99.70%	$-$	$-$
	3D-SBDD	−6.592$\pm$2.08	0.507$\pm$0.19	0.634$\pm$0.14	4.723$\pm$0.65	0.698$\pm$0.10	66.06%	$-$	$-$
	TargetDiff	−7.163$\pm$1.72	0.472$\pm$0.20	0.585$\pm$0.12	4.519$\pm$0.84	0.717$\pm$0.09	92.70%	100%	99.21%
	DiffSBDD	−7.214$\pm$1.91	0.461$\pm$0.21	0.579$\pm$0.13	4.571$\pm$0.88	0.729$\pm$0.07	96.45%	100%	99.92%
	InterDiff	-6.$812\pm$1.37	0.403$\pm$0.17	0.581$\pm$0.11	4.$242\pm$1.03	$0.769\pm$ 0.06	96.70%	100%	99.87%
DOCKSTRING	GraphBP	−6.85$\pm 1.79$	$0.464\pm$ 0.19	$0.499\pm$ 0.14	$4.416\pm$ 0.96	$\mathbf{0.789}\pm$ 0.02	99.73%	$-$	$-$
	Pocket2Mol	−7. 54$\pm 1.90$	$\mathbf{0.607}\pm$ 0.17	$\mathbf{0.722}\pm$ 0.13	$\mathbf{4.78}\pm$ 0.50	$0.678\pm$ 0.13	99.54%	$-$	$-$
	3D-SBDD	−7.32$\pm 1.57$	0.573$\pm$0.19	$0.578\pm$ 0.18	$4.713\pm$ 0.68	$0.559\pm$ 0.04	68.10%	$-$	$-$
	TargetDiff	−8.01$\pm 1.73$	$0.582\pm$ 0.18	$0.567\pm$ 0.12	$4.75\pm$ 0.55	$0.675\pm$ 0.06	96.53%	$-$	99.37%
	DiffSBDD	−7.93$\pm 1.76$	$0.561\pm$ 0.19	$0.548\pm$ 0.16	$4.73\pm$ 0.82	$0.696\pm$ 0.08	97.16%		99.87%
	InterDiff	−7.57$\pm 1.67$	$0.421\pm$ 0.21	$0.572\pm$ 0.18	$4.26\pm$ 01.22	$0.766\pm$ 0.10	97.42%	$-$	100%

To further assess the performance of different methods, we evaluate the distributions of molecular size and atom frequency, as illustrated in Figure S4. The molecules in train and test set are marked with red and mistyrose. The test set consists of a total of 97 non-repetitive molecules, which is fewer compared to the training set and the generated molecules, resulting in a more concentrated frequency distribution. In terms of molecular size, the density functions of molecules in the training set and the test set are very similar, and they exhibit a high degree of overlap with the distribution of molecules from the diffusion model. This can be attributed to the fact that the diffusion model needs to determine the size of the molecule before generation, usually by sampling based on the size of the pocket, while the autoregressive model predicts when to terminate during the molecule generation process, and the size of the molecule can only be determined after generation is completed. From Figure S4, we can see that InterDiff, DiffSBDD and TargetDiff have similar distributions in terms of molecule size and atomic frequency, which is also similar to the distributions in train set but differing significantly from the distribution of the autoregressive model. We observed that GraphBP tends to generate larger molecules, which may be related to differences in the selection criteria of its training set (only filtering conformations with RMSD below 2 Å), and GraphBP contains a greater variety of ligand atoms, up to 27 types. Additionally, we noticed that Pocket2Mol has a significantly different atomic frequency distribution compared to other methods, which may be related to its higher QED and other metrics. and we will investigate the reasons in future research.

#### Performance of InterDiff in design-specific interactions

**Figure 3:**
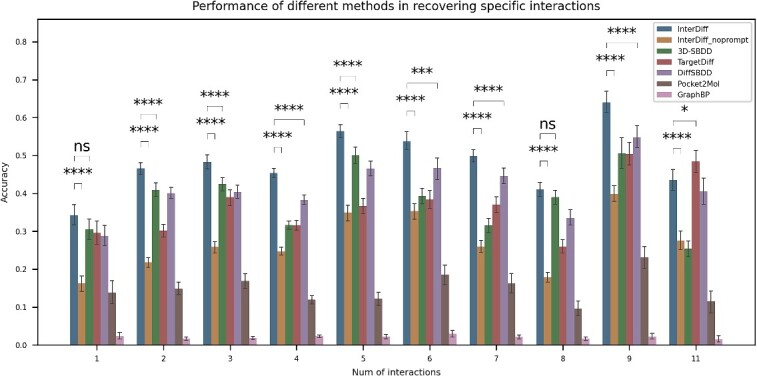
Accuracy of achieving designated interactions in generated molecules for different methods in CrossDocked2020 test set. Y axis denotes the accuracy, and X axis denotes the number of interactions detected in each protein–ligand complex. The statistical test is performed between InterDiff, InterDiff with no interaction information provided and the other baseline methods. ‘*’ indicates the significance level of the statistical test. ‘ns’ indicates that there is no significant difference between two groups of samples.

To evaluate the capability of InterDiff in generating molecules with designated interactions, we re-generate molecules in the CrossDocked2020 test set for 100 times. Additionally, we excluded the test samples that did not detect any interaction and 99 samples are left for testing. After generation, the interactions were detected by BINANA2 using the conformers generated by InterDiff. We computed the accuracy of accomplishing designated interactions for generated molecules. As an illustration, if *[‘GLU’, 14, ‘Hydrogen’]* (14th residue GLU with hydrogen bond) and *[‘TYR’, 39, ‘caption’]* are given as the condition for generating, and only *(‘TYR’, 39, ‘caption’)* is obtained in the generated molecular conformer, the accuracy would be 50%. The results are exhibited in Figure 3.

The number of interactions in the test set ranges from 1 to 11, with the exception of 10 interactions. We generate 100 samples for each protein pocket. Overall, InterDiff demonstrates excellent performance in designing molecules under specific interaction prompts, outperforming other methods, and 3D-SBDD ranks second (Figure 3). In order to further validate the role of interaction prompt, we also evaluated the generation results of InterDiff without providing any interaction prompt (InterDiff_noprompt in Figure 3). As shown in Figure 3, it can be observed that the accuracy of interaction restoration by InterDiff significantly decreases when no interaction prompt is provided, but still outperforms methods such as Pocket2Mol and GraphBP. In the absence of an interaction prompt, the ligand features obtained by the model during sampling tend to resemble the distribution of ligand features in the training set that do not involve interactions. This leads to the omission of interactions that require specific atomic and structural features, such as halogen interactions dependent on halogen atoms and π-π interactions dependent on aromatic ring structures. Furthermore, we observed that in the autoregressive methods, 3D-SBDD achieves significantly higher accuracy in restoring interactions compared to Pocket2Mol and GraphBP. This may be attributed to its simultaneous generation of coordinates and atomic types at each step of the generation process, effectively modeling the joint distribution of coordinates and atomic types, while Pocket2Mol and GraphBP separate the generation of coordinates and atomic types into two distinct steps. Therefore, we believe that modeling the joint distribution can better preserve global contextual information, enabling 3D-SBDD to perform comparably to diffusion models in terms of accuracy in restoring interactions. For InterDiff given interaction prompt, the highest accuracy is achieved under nine interactions and the lowest accuracy under one interaction. In cases with nine interactions, 5 molecules attain the same interaction types as the reference and 13 molecules are in accord with eight interactions. To verify the agreement between the generated conformers and the docking poses, we compare the raw conformers from InterDiff with the docking poses generated by QuickVina. We plot the resulting RMSD density distribution of nine conformers (ordered by the docking score from QuickVina) (Figure S6). For the besting scoring pose, QuickVina agrees with 9% of generated conformers (RMSD below 2 Å), which is similar to the work that Schneuing *et al.* [9] reported. Additionally, we also observe a significant drop of the proportion of agreed molecules in the less confident poses (4% in the 9th pose). Furthermore, to assess the ability of InterDiff in achieving disparate interactions, we evaluate the accuracy of four interactions in each sample and the results are presented in Figure S5.

Overall, InterDiff behaves distinctly in designing disparate interaction. The probability of hydrogen bonds being designed is the highest, followed by halogen bond. We note that InterDiff does not perform well in π-π interactions, which is understandable since the requirements of π-π interaction are more complicated than the others. Accordant with BINANA2’s criterion, three aromatic residues are involved. The distance between the aromatic ring center of the ligand and protein must be less than 4.4 Å. The ring atoms cannot deviate from planarity by more than 15 degrees. Lastly, the angle between two normal vectors in the ring planes must be within 30 degrees. Compared to other interactions, π-π interactions necessitate an aromatic ring structure in the ligand, which must be arranged appropriately. In addition, we noticed that the π-π interactions only comprise around 8% of the total interaction samples. The imbalanced interaction distribution may also impact the model performance. Last but not least, In the post-generation inference of molecular chemical bonds, the correct reconstruction of the aromatic ring structures within ligands is imperative. Therefore, the success rate of designing π-π interactions also hinges on the subsequent chemical bond inference algorithms. Explicitly incorporating chemical bonds into the generation process can enhance the accuracy of successfully designing this interaction.

#### Application of InterDiff in real scenarios

In this section, we investigate the potential of InterDiff in designing drugs when the binding mode of a reference molecule is available. The results demonstrate that InterDiff is capable of designing molecules based on specified interaction patterns. Specifically, we select two protein targets with different subtypes and use InterDiff to design molecules that exhibit a similar binding mode to existing drugs. The first target is muscarinic acetylcholine receptor (mAChR), acting as an important target in central nervous system diseases, for instance, Alzheimer’s disease and schizophrenia [28]. Xanomeline was developed as an agonist to mAChRs and studies have found that it has almost identical binding affinity to all mAChR subtypes (M1–M5), but stimulates them to an appreciably different extent [29]. A recent study revealed that the binding mode of xanomeline differs between inactive states and active states of mAChRs [30]. We employed InterDiff to rationally design compounds for M2-type mAChR in both inactive state and active state, conditioning on the binding mode of xanomeline. The second target is KRAS, frequently mutated in cancers and serving as a therapeutic targeting, such as lung cancer, colorectal cancer and pancreatic cancer. Current inhibitors only target KRAS G12C mutants, but the non-G12C mutants constitute the most in KRAS driven cancers. Recently, Kim *et al.* reported a non-covalent inhibitor BI-2865, which can bind to a wide range of KRAS altercations [31]. In like manner, we employed InterDiff to design novel compounds, leveraging the binding mode of BI-2865 in KRAS G12C and another mutant G13D as references. We generated 300 molecules for each state of two targets and evaluated the interactions after docking. The original interactions for two drugs are listed in Table S4. Among the generated molecules, we successfully obtained molecules that exhibit identical or improved interactions compared to the existing drugs. For illustrative purposes, we randomly select four molecules for illustration.

**Figure 4:**
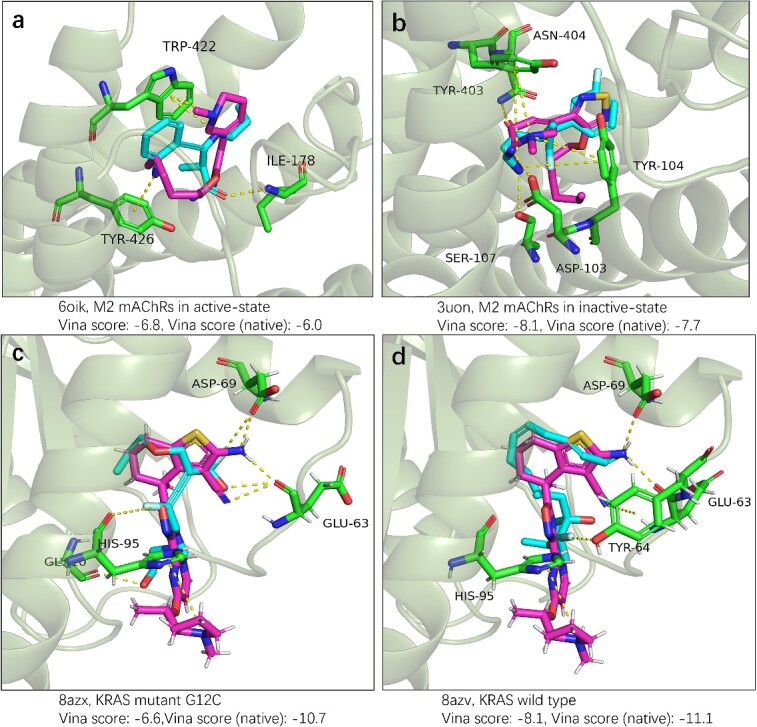
Pose of generated molecules and native drug in protein target (mAChR and KRAS protein). (**A**, **B**) The illustration of xanomeline and generated molecules in M2 mAChRs active state and inactive state. (**C**, **D**) Generated molecules and BI-2865 in KRAS wild type and G12C mutant. The binding poses of four generated molecules and Xanomeline are obtained by QuickVina, while the pose of BI-2865 is obtained from the cocrystal structure. Residues that have interactions with molecules are colored with green and labeled by abbreviations and their order in PDB file (for example, HIS-95 means 95th residue is histidine). BI-2865 and xanomeline are colored with purple, and generated molecules are colored with blue. The structures of all molecules are available in Figure S7.

Figure 4 displays the poses of generated molecules from docking in protein pockets, alongside the native targeted drugs. The structures of illustrated molecules are shown in Figure S7. The docking poses of xanomeline in mAChRs are acquired through docking, while the poses of BI-2865 in KRAS are obtained from the cocrystal structure in the PDB database. It is evident that the docking pose of designed molecules overlap well with the reference drugs. Additionally, InterDiff successfully generates similar interactions as the reference drugs in three of the four protein targets. In the last target (Figure 4D, KRAS wild type), three of the four interactions are consistent. InterDiff successfully designs molecules with better docking scores in both the active and inactive state of mAChRs. For the KRAS target, the docking scores are suboptimal compared to BI-2865 in the generated 300 molecules. Increasing the size or sampling times of molecules may help to discover molecules with better docking scores.

Figure 4A and B depict the docking pose of generated molecules and xanomeline for M2 mAChRs in both the active and inactive state. In the active state (PDB:6oik), the original interaction is TRP-422 with interaction cation-π. InterDiff successfully reproduces this interaction and introduces two new interactions, TYR-426 with cation-π and ILE-178 with hydrogen bond. For the inactive state (PDB:3uon), the primary interactions are cation-π in TYR-403 and TYR-104. InterDiff also replicates these interactions and forms three extra hydrogen bonds with ASN-404, SER-107and ASP-103. In the case of the KRAS mutant G12C cocrystal structure (PDB:8azx), BINANA2 discovers three interactions: ASP-69 with a hydrogen bond, GLU-63 with a hydrogen bond and HIS-95 with a cation-π interaction. While in KRAS wild type (PDB:8azv), an additional interaction, TYR-64 with cation-π is found. InterDiff discover molecules with a similar binding mode in KRAS mutant G12C (an extra interaction GLY-10 with hydrogen, Figure 4C) and KRAS wild type except for the interaction HIS-95 with cation-π.

#### Fragment growing by inpainting

In this section, we investigate the potential of InterDiff in fragment-based drug design (FBDD). Our findings demonstrate that InterDiff can design new fragments that interact with specified residues on existing fragments. It is often desirable to optimize certain parts of a molecule while keeping the scaffold intact. To this end, we also developed an unconditional diffusion model that learns the joint distribution of ligand atoms and protein atoms. The model structure and training process are similar to the conditional InterDiff, with the exception of the training objective that includes protein atoms in the loss function. To generate molecules based on a given scaffold, we modify the sampling process by injecting the fixed context in the denoising steps and replacing the corresponding parts from the model. This technique, known as inpainting, was initially introduced in image imputation [15, 32]. Formally, in each denoising step, we do the following operations:


$$ {x}_{t-1}^{known}\sim \mathcal{N}\left({x}_t|\sqrt{1-{\beta}_t}{x}_0,{\beta}_t\boldsymbol{I}\right), $$

$$ {x}_{t-1}^{unknown}\sim \mathcal{N}\left({\overset{\sim }{\mu}}_{\theta}\left({x}_t\right),{\overset{\sim }{\beta}}_t\boldsymbol{I}\right), $$

$$ {x}_{t-1}=m\bigodot{x}_{t-1}^{known}+\left(1-m\right)\bigodot{x}_{t-1}^{unknown}, $$

where ${x}_{t-1}^{known}$ indicates the reference samples, ${x}_{t-1}^{unknown}$ indicates the samples from the model and $m$ is a binary mask that signifies the fixed context. In the experiments, the pocket atoms and the native ligand are the ${x}_{t-1}^{known}$ and the denoising samples from the model are ${x}_{t-1}^{unknown}$.

**Figure 5:**
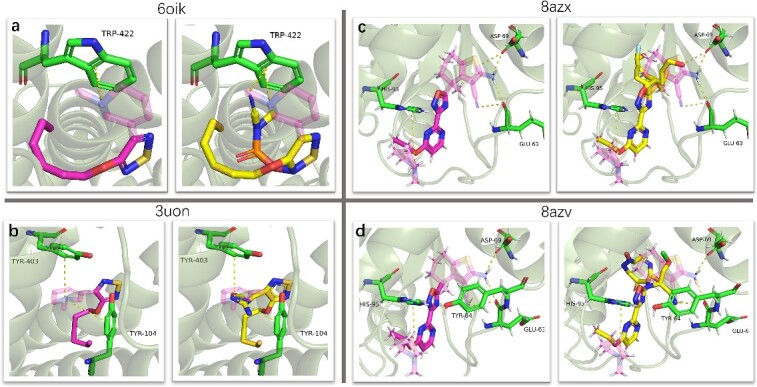
Pose of designed fragments with scaffold and native drug in the protein target. The transparent parts of native drug are fragments with atomic interactions. Residues that have interactions with molecules are colored with green and labeled by abbreviations and their order in PDB file (for example, in subfigure a, TRP-422 means 422nd residue is tryptophan). BI-2865 and xanomeline are colored with purple, and generated molecules are colored with yellow.

In this experiment, we utilized the same protein targets as in the previous section and sampled 100 molecules for each state of the two targets. We evaluate InterDiff in FBDD by removing the fragments (Figure 5, transparent parts) that have interactions with protein residues, while retaining the remaining molecules as fixed scaffolds. Four illustrative examples are shown in Figure 5, and we successfully design fragments with the same interactions as the native drug in M2 mAChRs based on the scaffold. For the KRAS mutant G12C (PDB:8azx) and KRAS wild type (PDB:8azv), one (ASP-69 with hydrogen) of three and two (ASP-69 with hydrogen, TYR-64 with cation-π) of four interactions are achieved. The docking poses are generated by the model, and our method effectively inpainted new fragments with desired interactions around the fixed scaffolds. Nonetheless, we observed that the accuracy of InterDiff in inpainting mode is inferior to the pocket-conditional mode. The model must estimate the positions of both protein and ligand atoms in the denoising steps. On the contrary, only ligand atoms are estimated in the pocket-conditional mode. The errors in estimating protein atom types and locations could potentially impact the accuracy of ligand atom estimation. In addition, in case PDB:6oik, we discovered that the new fragments are anchored in an alternative position on the five-membered ring. It would be interesting to incorporate anchor point information into the diffusion model and generate diverse molecules. Overall, our findings demonstrate the efficacy of InterDiff in designing fragments with desired interactions, although improvements are needed to enhance the accuracy of the inpainting mode and consider anchor point information for generating diverse molecules.

### Related work

#### Diffusion model for molecular design

Diffusion models are a new kind of generative model inspired by diffusion process. Impressive progresses have been made in distinct generating task such as images, audios and even videos [33–35]. In molecular science, Hoogeboom *et al.* [36] first proposed the E(3) Equivariant Diffusion Model (EDM) for molecular generation, which notably outperforms previous 3D generative methods. Shortly after their work, Schneuing developed a diffusion model for structure-based drug design named DiffSBDD, which is the first of its kind [9]. Two strategies are introduced under their framework, protein-conditional and ligand-inpainting generation. Specifically, ligand-inpainting methods learn the joint distribution of protein–ligand complex, and new ligands are completed in inference stage. Experiments exhibit that both strategies can produce novel and drug-like ligands. *In silico* docking assessment also verifies the potential in generating ligands with high binding affinity. Similar work was done in Huang [13], and the difference lies in the fact that dual diffusion was used to capture the local and global protein environment. In addition, Guan *et al.* presented a target-aware diffusion model. Unlike previous work that needs to evaluate generated molecules through a docking method like AutoDock, their model can estimate and rank the binding affinity of molecules. The authors raise a problem that the bond inference is implemented in a post-processing manner and irrational structures may appear in generated molecules, which is also pointed out in Schneuing’s work [9]. Huang *et al.* [12] tackle this problem by setting distance threshold for covalent bond, but the bond distance can vary depending on the particular chemical structure. Alternatively, Wu *et al.* [37] developed a diffusion model guided by a prior diffusion bridge, which can guarantee a desirable output.

#### Prompt learning for molecular design

Prompt-based learning is initially a strategy to train large language models (LLMs), serving as an alternative to the fine-tuning paradigm so the LLMs can adapt to different tasks without re-training [38]. Afterward, this technique was introduced to the vision-language model and greatly improved the performance over all evaluation tasks [39]. Very recently, several attempts have been made to incorporate prompt-based learning to molecular design [40–43]. These works combine SMILES representation of molecules with other modalities including chemical structure texts [40, 41], pharmacological properties [41–43], medical description texts [42] and protein pocket [43]. Gao *et al.* [43] propose a unified model called PrefixMol considering both chemical properties and binding pocket via generative pre-trained transformer (GPT). The pocket information is transformed into an embedding by geometric vector transformer (GVF) and used as a prefix condition together with other conditional embeddings. PrefixMol demonstrates excellent performance in single and multiple conditional molecular generation. But still, PrefixMol is an autoregressive model, and the global context of ligands are lost during the generation process. Moreover, their model treats pocket residues equally and the outputs are 1D SMILES representation, which makes it hard to apply in real scenarios.

## DISCUSSION

In this work, we introduce a novel molecular diffusion generative model named InterDiff by residue interaction prompt. Our model has the capability to generate molecules that have specific interactions with designated residues, with both high statistical probability and high binding energies. This ability is critical for structure-based drug design, as it can significantly enhance the efficiency of drug development and provide valuable design insights. On the other hand, we demonstrate that InterDiff could be easily adapted into a fragment-based generative model, enabling optimization molecules by incorporating interactions with certain hotspot residues. The results show that InterDiff could generate new fragments that interact with hotspots based on a molecular scaffold. This characteristic is of wide interests for modern *in silico* drug design. Nevertheless, InterDiff still faces challenges such as the unsynthesizable structures presented in the generated molecules, which are also commonly seen in other molecular generative methods. While this may be attributed to flaws in molecular reconstruction algorithms, it is imperative to make continuous efforts to improve the rationality of the generated molecular structures. In our future work, we aim to optimize the sub-structures of generated molecules to ameliorate the drug-likeness and synthetic accessibility. One approach we will explore is incorporating chemical bond information into the training and generation process. While this has the potential to address the aforementioned issues, it may also introduce increased complexity in model training. In addition, improving accuracy in designing π-π interactions could be important in future optimization. π-π interaction requires the generation of the correct aromatic ring structure, which demands a model that can accurately generate the positions of each atom and determine the chemical bond information between atoms. Therefore, for SBDD, a diffusion generation model is needed that can generate a complete 3D molecular structure, including coordinates, atom types and chemical bond information. Currently, the selection of the interaction prompts for residues primarily relies on the reference molecules or experience of pharmaceutical chemists. Existing tools like FTMap may be helpful to solve this problem [44].

## METHODS

### Molecular diffusion model

We build our model upon the framework develop by Guan *et al*. [10]. Let $\mathcal{M}=\left(x,h\right)$ denote the molecular 3D point cloud data with $x=\left[{x}^{(L)},{x}^{(P)}\right]\in{\mathcal{R}}^{N\times 3}$ and $h=\left[{h}^{(L)},{h}^{(P)}\right]\in{\mathcal{R}}^{N\times M}$. In our setting, $\left[{x}^{(L)},{x}^{(P)}\right]$ indicates atom coordinates of ligand and protein, and $\left[{h}^{(L)},{h}^{(P)}\right]$ represents the atom categorial features, where $N$ is the number of atoms and $\left(\cdot \right)$ marks the scripts that are not index. For example, $(L)$ indicates ligand and $(P)$ indicates protein. We use the diffusion model to learn the distributions of protein–ligand complexes. The diffusion model learns two Markov processes, a diffusion process $q$ and a denoising process $p$. The diffusion process adds Gaussian noise to data ${\mathcal{M}}_t$ in time step $t$, where $t=0,\cdots, T-1$ is the predefined time steps ($T=1000$ in the implementation):


$$ q\left({\mathcal{M}}_t|{\mathcal{M}}_{t-1}\right)=\mathcal{N}\left({\mathcal{M}}_t|{\alpha}_t{\mathcal{M}}_{t-1},{\sigma}_t^2\boldsymbol{I}\right), $$

where ${\alpha}_t$ is the schedule that controls how much signals are preserved in the diffusion process and ${\sigma}_t$ is the noise schedule that controls how much noises are added. For the 3D molecular point cloud data, the atom types are categorical data while the atom coordinates are continuous data. At time step $t$, we add Gaussian noise and uniform noise to the atom coordinate features and atom type features, respectively [45]. Following the convention in Guan *et al*. and Hoogeboom *et al*. [10, 45], the joint distribution states as:


$$ q\left({\mathcal{M}}_t|{\mathcal{M}}_{t-1}\right)=\mathcal{N}\left({x}_t|\sqrt{1-{\beta}_t}{x}_{t-1},{\beta}_t\boldsymbol{I}\right)\cdot \mathcal{C}\left({h}_t|\left(1-{\beta}_t\right){h}_{t-1}+{\beta}_t/K\right), $$

where $\mathcal{C}$ indicates a categorical distribution with parameters after $\mid$, ${\beta}_t$ is the variance schedules and $K$ is the number of atom types with $k=1,\cdots, K$. In the implementation, the variance is reduced as the steps grow. For the generative denoising process, the posterior distribution $p\left(\cdot \right)$ can be computed in a close form by the Bayesian formula:


$$ p\left({x}_{t-1}|{x}_t,{x}_0\right)=p\left({x}_t|{x}_{t-1},{x}_0\right)\frac{p\left({x}_{t-1}|{x}_0\right)}{p\left({x}_t|{x}_0\right)}=\mathcal{N}\left({x}_{t-1}|{\overset{\sim }{\mu}}_t\left({x}_t,{x}_0\right),{\overset{\sim }{\beta}}_t\boldsymbol{I}\right), $$

$$ p\left({h}_{t-1}|{h}_t,{h}_0\right)=p\left({h}_t|{h}_{t-1},{h}_0\right)\frac{p\left({h}_{t-1}|{h}_0\right)}{p\left({h}_t|{h}_0\right)}=\mathcal{C}\left({h}_{t-1}|{\boldsymbol{\theta}}_{post}\left({h}_t,{h}_0\right)\right), $$

where ${\boldsymbol{\theta}}_{post}\left({h}_t,{h}_0\right)=\overset{\sim }{\boldsymbol{\theta}}/{\sum}_{k=1}^K{\overset{\sim }{\boldsymbol{\theta}}}_k$, $\overset{\sim }{\boldsymbol{\theta}}=\left[{\alpha}_t{h}_t+\left(1-{\alpha}_t\right)/K\right]\odot \left[{\overline{\alpha}}_{t-1}{h}_0+\left(1-{\overline{\alpha}}_{t-1}\right)/K\right]$, ${\overset{\sim }{\mu}}_t\left({x}_t,{x}_0\right)=\frac{\sqrt{{\overline{\alpha}}_{t-1}}{\beta}_t}{1-{\overline{\alpha}}_t}{x}_0+\frac{\sqrt{\alpha_t}\left(1-{\overline{\alpha}}_{t-1}\right)}{1-{\overline{\alpha}}_t}{x}_t$, ${\overset{\sim }{\beta}}_t=\frac{1-{\overline{\alpha}}_{t-1}}{1-{\overline{\alpha}}_t}{\beta}_t$ and ${\alpha}_t=1-{\beta}_t$, ${\overline{\alpha}}_t={\prod}_{s=1}^t{\alpha}_s$. In the denoising process, ${x}_0$ and ${h}_0$ are approximated by neural network, and we denote the approximation of ${x}_0$ and ${h}_0$ as ${\hat{x}}_0,{\hat{h}}_0={\Phi}_{\Omega}\left({x}_t,{h}_t,t\right)$, where $\Phi$ is a neural network parameterized by $\Omega$. The training objective is the summation for atom coordinates and atom types. The atom coordinate loss states as:


$$ {L}_{t-1}^x={\gamma}_t{\left\Vert{x}_0-{\hat{x}}_0\right\Vert}^2+C, $$

where ${\gamma}_t$ is the weight for MSE loss and $C$ is a constant. In the implementation, we set ${\gamma}_t=1$ for all time steps. The atom type loss is computed by Kullback-Leibler divergence of two categorical distributions:


$$ {L}_{t-1}^h={\sum}_k{\boldsymbol{\theta}}_{post}{\left({h}_t,{h}_0\right)}_k\cdot \mathit{\log}\frac{{\boldsymbol{\theta}}_{post}{\left({h}_t,{h}_0\right)}_k}{{\boldsymbol{\theta}}_{post}{\left({h}_t,{\hat{h}}_0\right)}_k}. $$

The final loss is calculated by the weighted summation of MSE loss, KL-divergence and a classification loss:


$$ L={\lambda}_x{L}_{t-1}^x+{\lambda}_h{L}_{t-1}^h+{\lambda}_c Cls\left({h}^{(p)}\right), $$

where ${\lambda}_x$, ${\lambda}_h$ and ${\lambda}_c$ are the weight for MSE loss, KL-divergence and classification loss, respectively, and ${h}^{(p)}$ indicates the protein atom features. The classification loss classifies the protein atom features into different interaction types, and the cross-entropy are used in the experiment.

#### Equivariant diffusion under prompt guidance

In this section, we elaborate our proposed InterDiff model. InterDiff is a graph neural network in which the atoms denote nodes and the Euclidean distance between atoms denote edges. We define an edge among two nodes when the Euclidean distance is below 7 Å. Let $\boldsymbol{v}=\left({v}_I^{(d)},{v}_I^{(c)}\right)$ denote the interaction prompts, where ${v}_I^{(d)}\in{\mathcal{R}}^4$ are one-hot representation prompts, $I\in \left( cation- pi, halogen, hydrogen, pi- pi\right)$ indicate the interaction types and ${v}_I^{(c)}$ are learnable continuous embeddings. The superscript $(d)$ and $(c)$ indicate two types of prompts (one-hot/discrete and continuous). The atom node features are also encoded by one-hot vectors and transformed by a single linear layer: ${h}^{0,(p)}= Linear\left(\left[{h}^{(P)},{v}_I^{(d)}\right]\right),{h}^{0,(L)}= Linear\left({h}^{(L)}\right)$, where ${h}^{0,(P)}$ and ${h}^{0,(L)}$ represent the protein and ligand atom features in the $0$th layer (before the six equivariant block layers).

**Figure 6:**
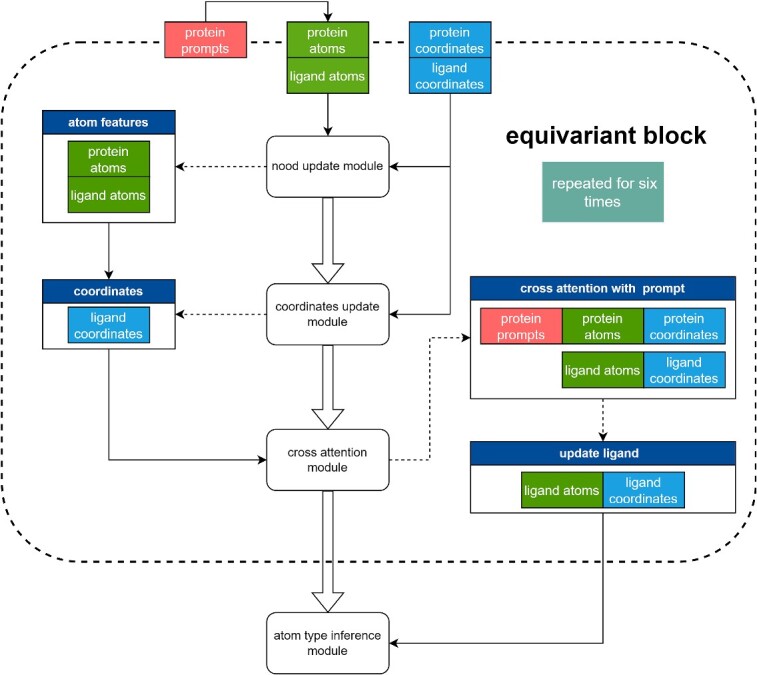
Structure of equivariant block used in InterDiff. Modules are represented by rounded rectangle with white context, while data are shown by rectangle with distinct colors. Input and output flows are shown with arrowhead and dashed arrowhead, respectively.

InterDiff is composed of six equivariant block layers (Figure 6), and each block consists of three modules. Formally, the first module updates the node features:


$$ {h}^{l-1,(P)}={h}^{l-1,(P)}+{v}_I^{(c)}, $$

$$ {m}_{i,j}= cat\left({d}_{ij,}{h}_i^{l-1},{h}_j^{l-1}\right), $$

$$ {h}_k= MLP\left({m}_{i,j}\right),{h}_v= MLP\left({m}_{i,j}\right),{h}_q= MLP\left({h}^{l-1}\right), $$

$$ {h}^l={h}^{l-1}+ attention\left({h}_k,{h}_q,{h}_v\right), $$

where ${h}^{l,(P)}$ indicates protein atom features in $l$th layer, ${d}_{ij}$ is the Euclidean distance between atom $i$ and $j$,${m}_{i,j}$ is the concatenation of pairwise protein and ligand atom features and Euclidean distance. $cat\left(\cdot \right)$ indicates the concatenation operation and $MLP\left(\cdot \right)$ is the multilayer perceptron. The second module updates the ligand coordinates:


$$ {m}_{i,j}= cat\left({d}_{ij,}{h}_i^{l-1},{h}_j^{l-1}\right), $$

$$ {h}_k= MLP\left({m}_{i,j}\right),{h}_v= MLP\left({m}_{i,j}\right),{h}_q= MLP\left({h}^{l-1}\right), $$

$$ {h}_v={h}_v\cdot{\left[{x}_i^{l-1}-{x}_{j,j\in Ne(i)}^{l-1}\right]}_{i=1}^N $$

$$ {x}^{l,(L)}={x}^{l-1,(L)}+ attention\left({h}_k,{h}_q,{h}_v\right), $$

where ${x}^{l,(L)}$ indicates ligand atom coordinates in the $l$th layer, ${x}_{j,j\in Ne(i)}^l$ represents that $j$th atom coordinates and $j$ is the neighbors of atom $i$. The third module updates ligand atom features and coordinates simultaneously with cross attention:


$$ {\tilde{d}}_{ij}= dis\_ encoding\left({d}_{ij}\right) $$

$$ cxt= cat\left({h}^{l_h,(P)},{v}_I^{(c)}\right) $$

$$ {h}^{l,(L)},{x}^{l,(L)}= crossatte\left( cxt,{\tilde{d}}_{ij},{d}_{ij},{h}^{l_h,(L)},{x}^{l_x,(L)},{x}^{l_x,(P)}\right), $$

where $dis\_ encoding\left(\cdot \right)$ is the encoding of the distance matrix between ligand and protein and ${h}^{l_h,(L)}$, ${h}^{l_h,(P)},{x}^{l_x,(L)}$ and ${x}^{l_x,(P)}$ indicate the node features and coordinates of ligand and protein from the first and second module. Superscript ${l}_h$ and ${l}_x$ indicate the output from first and second module in the $l$th layer. For the distance encoding, we use multilayer perceptron in the implementation. The $crossatte\left(\cdot \right)$ is computed as follows:


$$ {h}_{qk}^{l,(L)}= Linear\left({h}^{l_h,(L)}\right),{cxt}_{qk}= Linear(cxt), $$

$$ sim= MLP\left( cat\left({h}_{qk}^{l,(L)}\cdot{cxt}_{qk},{\tilde{d}}_{ij}\right)\right), $$

$$ {\tilde{h}}^{l,(L)}= softmax(sim)\left[:\frac{1}{2} nheads\right]\cdot{cxt}_v, $$

$$ {\tilde{x}}^{l,(L)}= mean\left( softmax(sim)\left[\frac{1}{2} nheads:\right]\right), $$

$$ {h}^{l,(L)}={h}^{l_h,(L)}+ MLP\left({\tilde{h}}^{l,(L)}\right), $$

$$ {x}^{l,(L)}={x}^{l_x,(L)}+{\left[{x}_i^{l_x,(L)}-{x}_j^{l_x,(P)}\right]}_{ij}\cdot MLP\left({\tilde{x}}^{l,(L)}\right), $$

where $nheads$ is the number of heads in cross-attention mechanism and $sim$ is the attention map between ligand and protein. Note that we ignore the operations of dimension rearrangement in the above formulas and simplify Einstein summation to dot product here. Molecules are subject to geometric symmetries in 3D space, such as translations and rotations. These symmetries are known as the Euclidean group in three dimensions, denoted as SE(3). Studies have found that extending translation and rotation symmetries in molecular systems improves generalization [46]. In diffusion generative models, the SE(3)-equivariance of Markov transition is a characteristic that refers to the invariance of the Markov transition probability matrix under the action of the special Euclidean group SE(3). Identical to previous work [36, 47, 48], we use ‘subspace-trick’ by limiting the center of mass (CoM) of training samples to zero to ensure the model can achieve translation invariance in the generative process. For the SE(3)-equivariance of Markov transition, the proof of the first two modules is similar to Guan *et al*. [10] and we prove the equivariance for the cross-attention module in the supplementary.

#### Ablation study

In order to assess the impact of proposed module of our model, we remove the proposed cross-attention module in each equivariant block and train the model to carry out the ablation study in the CrossDocked2020 dataset. We detect the docking score and accuracy of recovering specific number of interactions in generated molecules. The results are shown in Table S2, which illustrates the docking score and accuracy of recovering different numbers of interactions of InterDiff and InterDiff-r (with no cross-attention modules). In terms of docking scores, the removal of the cross-attention module did not have a significant impact, only resulting in a slight decrease of approximately 0.1, with no statistical difference observed. However, the experiments results illustrate that the accuracy of recovering specific number of interactions (2, 5, 6, 7, 8) decrease significantly (Table S2). For samples containing five interactions and six interactions respectively, the accuracy of InterDiff-r decreased from 56% to 48% and from 54% to 47%. For interactions (3, 9, 11), InterDiff-r is slightly better than InterDiff and the largest difference is 0.66 versus 0.64 for nine interactions.

Overall, the ablation study results emphasize the significance of the cross-attention module in our model. We believe that compared to the message propagation mechanism on a graph within a specific distance, the cross-attention module can capture long-range interactions and guide the design of molecules, ultimately leading to improved performance.

**Key Points**

- Compared with autoregressive-based generative models, diffusion-based generative models sample ligand coordinates and atom types simultaneously in protein pockets, which avoid the accumulation of errors in autoregressive models.
- Hotspot residues are critical for drug efficacy and contribute most to the binding free energies. Our method first introduces the atomic interactions of hotspots into the molecular generative models, which is an important challenge in artificial intelligence–based drug design.
- This manuscript proposes a method for atomic interaction–based drug design and validates the model performance in two real protein targets. Our model can design molecules with a similar or better binding mode compared with native drugs.
- InterDiff can be modified into a fragment-based generative model and could generate new fragments that interact with hotspots given a molecular scaffold.

## Supplementary Material

Supplementary_file_bbae174

## Data Availability

CrossDocked2020 can be obtained at https://bits.csb.pitt.edu/files/crossdock2020/; structured models used in studies are deposited in Protein Data Band with accession codes 6oik, 3uon, 8azx and 8azv. The source code, sampling molecular conformers and notebook are available on GitHub: https://github.com/zephyrdhb/InterDiff.

## Author Biographies

**Peng Wu** is a PhD student in Shenzhen University.

**Huabin Du** is an Engineer in MoMed Biotechnology.

**Yingchao Yan** is an Engineer in MoMedBiotechnology.

**Tzong-Yi Lee** is a professor in National Yang Ming Chiao Tung University.

**Chen Bai** is a professor in The Chinese University of Hong Kong, Shenzhen and founder of MoMed Biotechnology.

**Song Wu** is a professor in Shenzhen University and director of South China Hospital.
