# Supplementary material for: Guided diffusion for molecular generation with interaction prompt
Source: Brief Bioinform. 2024 Apr 21;25(3):bbae174. doi: 10.1093/bib/bbae174 (PMC11033848; doi:10.1093/bib/bbae174)
Supplement: Supplementary_file_bbae174 [file supplementary_file_bbae174.docx]

# Supplementary

## Additional figures and tables

| Ring | GraphBP | Pocket2Mol | 3D-SBDD | TargetDiff | DiffSBDD | InterDiff |
| --- | --- | --- | --- | --- | --- | --- |
| 3 | 53.5% | 0.2% | 30.9% | 0.0% | 0.0% | 0.0% |
| 4  5 | 19.5%  9.8% | 0.1%  16.7% | 0.8%  20.2% | 4.2%  32.4% | 5.8%  30.6% | 10.5%  23.4% |
| 6 | 8.2% | 77.6% | 43.8% | 42.2% | 43.4% | 40.6% |
| 7 | 4.9% | 3.9% | 2.3% | 15.9% | 14.8% | 17.5% |
| 8 | 2.7% | 1.2% | 1.5% | 4.2% | 3.5% | 5.7% |
| 9 | 2.4% | 0.3% | 0.5% | 1.2% | 1.9% | 2.3% |

Table S1：Percentage of ring sizes of InterDiff and baseline methods in CrossDocked2020 test set.

|  | Docking score | 1 | 2 | 3 | 4 | 5 | 6 | 7 | 8 | 9 | 11 |
| --- | --- | --- | --- | --- | --- | --- | --- | --- | --- | --- | --- |
| InterDiff | 6.812$\pm$1.37 | **0.34** | **0.47** | 0.48 | **0.45** | **0.56** | **0.54** | **0.50** | **0.41** | 0.64 | 0.44 |
| InterDiff-r | 6.712$\pm$1.43 | 0.33 | 0.42 | **0.49** | 0.42 | 0.48 | 0.47 | 0.45 | 0.37 | **0.66** | **0.45** |

Table S2：Results of ablation study of InterDiff and baseline methods in CrossDocked2020 test set. ‘-r’ denotes the removal of cross attention module. The ‘Docking score’ column gives the average docking score of generated molecules. The columns from 1 to 11 indicate different number of interactions in the test set and the accuracy of recovering original interactions in the generated molecules.

| Parameter name | Value | Description |
| --- | --- | --- |
| hydrogen_bond_dist_cutoff | 3.2 Å | Donor/acceptor distance cutoff for hydrogen bonds |
| hydrogen_halogen_bond_angle_cutoff | 40.0° | Donor/acceptor distance and angle cutoff, in degrees, for hydrogen and halogen bonds |
| halogen_bond_dist_cutoff | 5.5 Å | Donor/acceptor distance cutoff for halogen bonds |
| pi_pi_interacting_dist_cutoff | 4.4 Å | Ring-center distance cutoff for detecting pi-pi stacking and T-shaped interactions |
| pi_stacking_angle_tolerance | 30.0° | pi-pi stacking angle cutoff, in degrees |
| t_stacking_angle_tolerance | 30.0° | pi-pi T-shaped angle cutoff, in degrees |
| t_stacking_closest_dist_cutoff | 5.5 Å | Atom-atom distance cutoff for detecting pi-pi T-shaped interactions |
| cation_pi_dist_cutoff | 6.6 Å | Charged-moiety/ring-center distance cutoff for cation-pi interactions |

Table S3：Summary of parameters of BINANA2 in identifying four interactions.


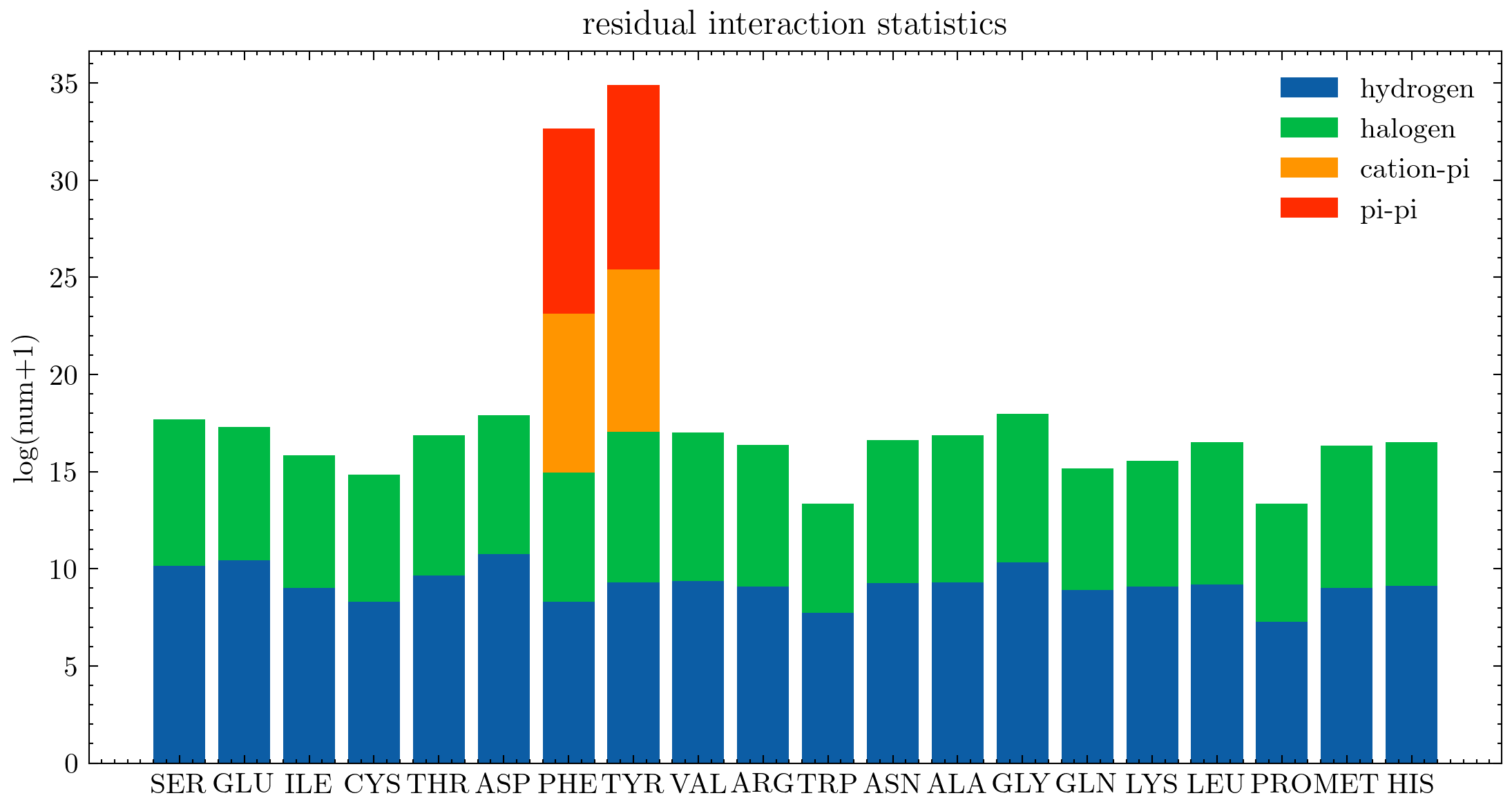


Figure S1: A bar plot of interactions in different protein residues. Y axis is illustrated with logarithmic scale to balance the height of the bar.


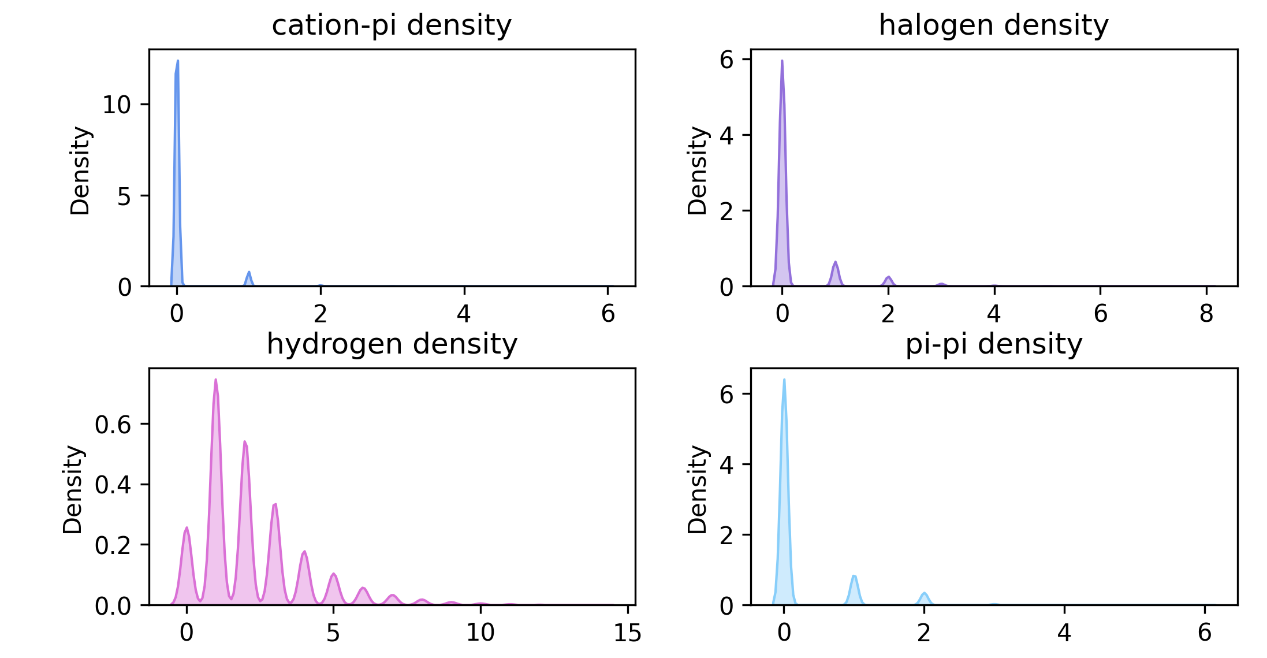
Figure S2: Density distributions of interactions in CrossDocked 2020 training set. The number of four interactions were count for each protein-ligand complex.


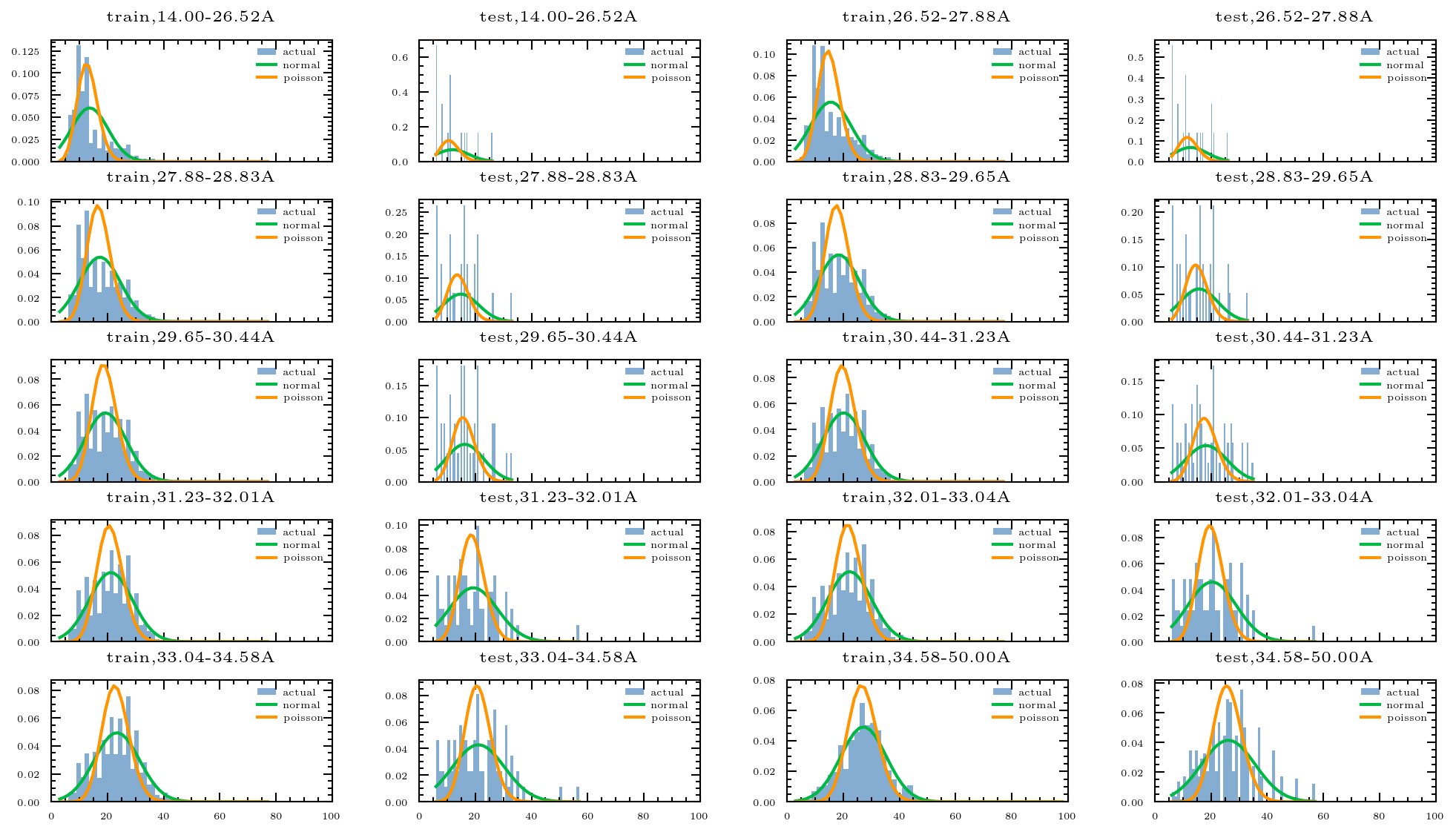


Figure S3: Distributions of molecular size in different pocket sizes. Pocket sizes are defined by the median distance of the 10 farthest of pocket atoms. We divide the pocket sizes range into 10 quantiles and plot the distributions of number of ligand atoms of each bin in training set and test set. There is a clear trend that the larger the pocket size is, the more the number of ligand atoms will have. In addition, we can see that the distribution is similar for the training and test set in each bin. The distribution in training set can be generalized to test set.


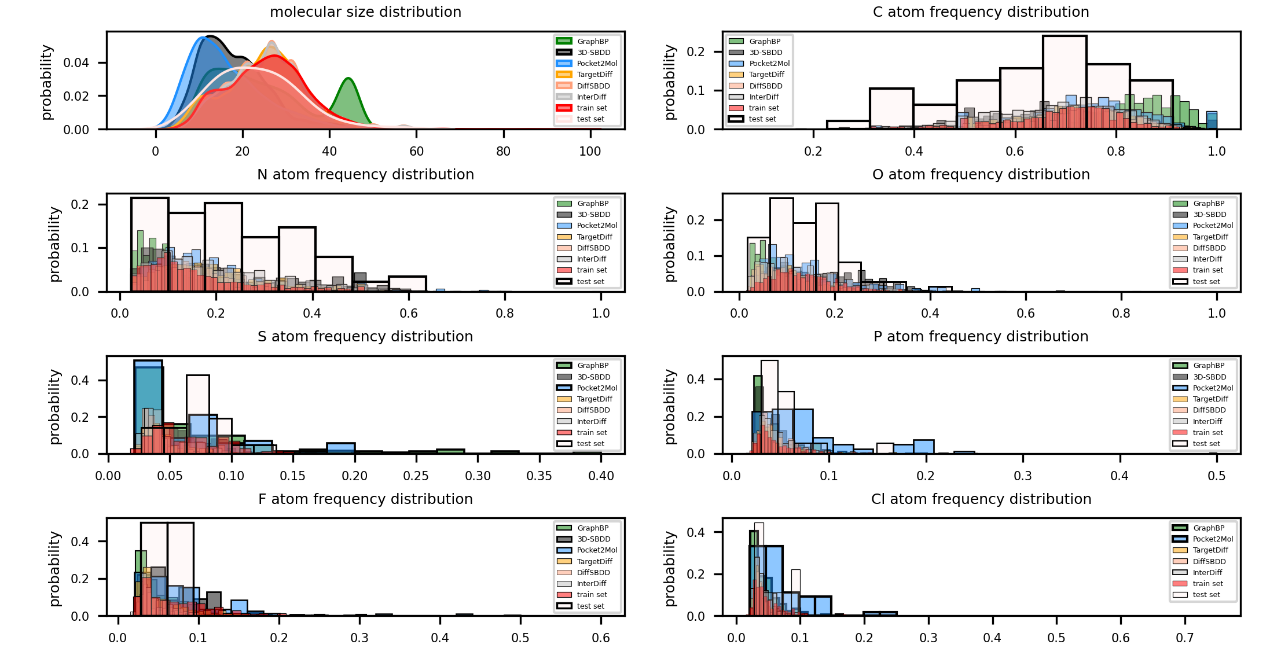


Figure S4: The size distribution of molecules generated by different methods and the molecules in train and test set, as well as the frequency distribution of different types of atoms in each molecule.


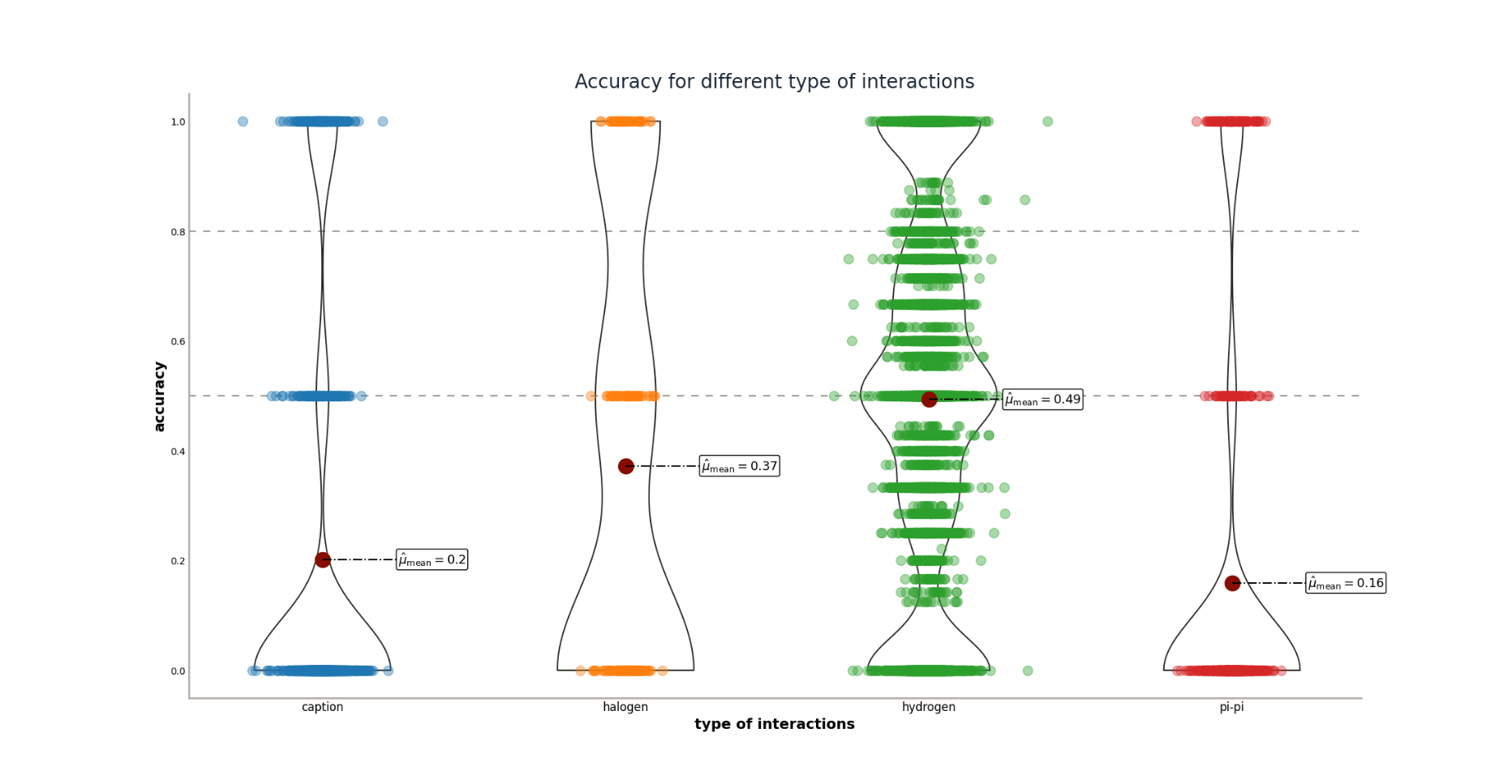


Figure S5: Accuracy of InterDiff in designing four types of interactions in test set.


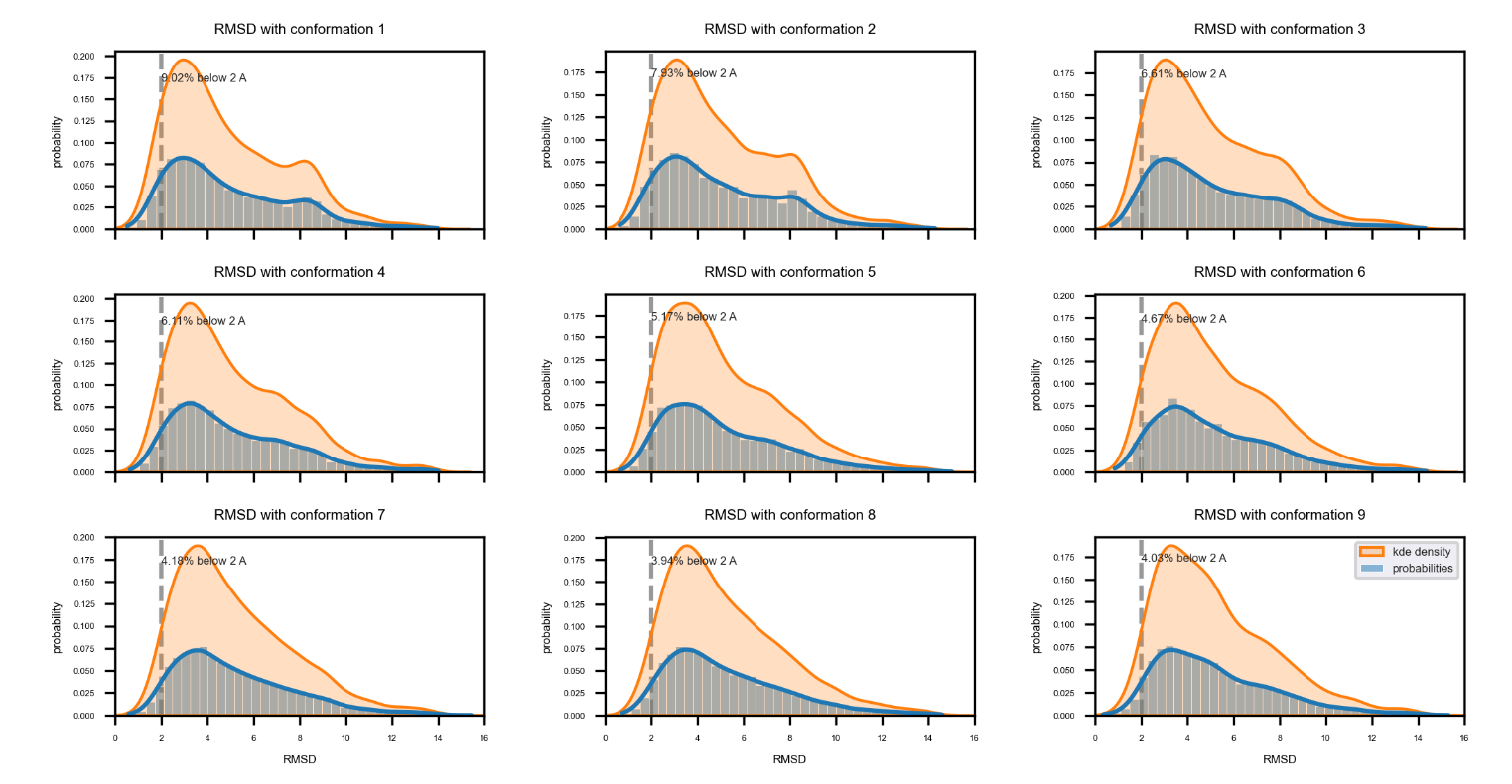


Figure S6: RMSD between original conformations generated by InterDiff and docked conformations in test set. 9 conformations are generated by QuickVina for each molecule and the percentage of RMSD below 2 angstrom is calculated.


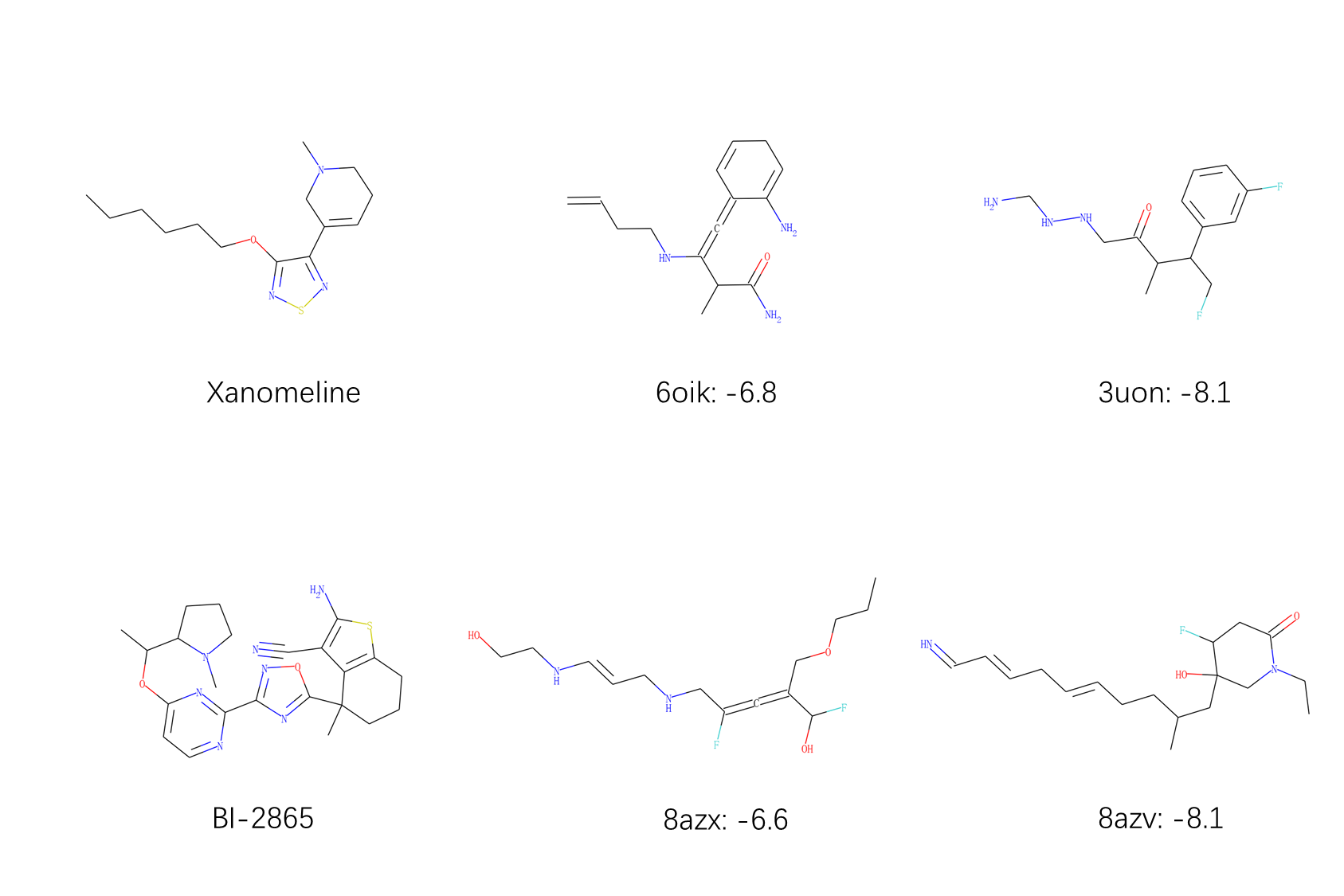


Figure S7: Native drugs and generated molecules for two protein targets, mAChR (top row) and KRAS (bottom row). The docking pose are illustrated in figure 4 and figure 5. Generated molecules are labeled with PDB accession codes and vina scores.

| PDB code | Target | interaction | Description |
| --- | --- | --- | --- |
| 6oik | mAChR active state | [(TRP,422,cation-π)] | The interactions are obtained by docking with QuickVina |
| 3uon | mAChR in-active state | [(TYR,104,cation-π),(TYR,403,cation-π)] | The interactions are obtained by docking with QuickVina |
| 8azx | KRAS mutant G12C | [(GLU,63,hydrogen),(ASP,69,hydrogen), (HIS,95,cation-π)] | The interactions are obtained from cocrystal structure |
| 8azv | KRAS wild type | [(GLU,63,hydrogen),(ASP,69,hydrogen), (TYR,64,cation-π),(HIS,95,cation-π)] | The interactions are obtained from cocrystal structure |

Tab S4: Interactions of targeting drugs in two protein targets.

### Experimental details

### Training and sampling

InterDiff consists of 6 equivariant blocks and each block has three modules with transformer like structure. The diffusion steps are set to 1000 in training and sampling. We utilize an adaptive cosine scheduler for atom coordinates and atom types[1], to control the rate at which the noise is added:

$$\alpha_{t}=\cos(\frac{\pi}{2}\frac{(\frac{t}{T}+s)^{\nu}}{1+s})^{2},$$

where the parameter $\nu$ can be set to different value for atom types and coordinates. In the experiment, we set $\nu$ to 2 for coordinates and 1 for types. The number of heads is 16 for the first two modules and 32 for the cross-attention module. The dimension is 128 for the atom features and interaction prompt $v_{I}^{(c)}$. We use Lion[2] method to optimize the model with an initial learning rate 0.0001, weight decay 1e-2 and the batch size is set to 8. A ‘plateau’ scheduler was applied to decay the learning rate with a factor 0.8 when the validation performance is stuck for 4 evaluation steps. The minimum learning rate is 1e-6. The loss weight is 100 for atom type loss and 1 for MSE loss and classification loss. We train InterDiff on one NVIDIA V100S GPU and the model converges within 32 hours. In addition, we empirically found that the model could be further improved on validation set (randomly selected from training data for validation) when fix the prompt embedding and fine tune after convergence.

The input data are processed from CrossDocked2020 dataset, which contains 22.5 million poses of ligands docked into multiple similar binding pockets across Protein Data Bank database. We use the protein-ligand complex with an RMSD below 1 Å to train our model. BINANA2 was used to detect the interactions in complex and we save the interaction information for all the protein residues. Four kinds of interactions are included in experiment as described in introduction part. We use learnable embeddings to represent these interactions and all embeddings are random initialized before training. The dimensionality of embedding is set to 128. In the training process, the interaction is given for each protein residue and the atoms in the same residue share the same interaction. The protein and ligand are expressed as graphs, which are atom types and atom coordinates.

In sampling process, the interaction prompts for each sample are provided in keeping with the molecule in test set. The center of mass is subtracted from the coordinates of protein atoms and the number of atoms is sample according to the pocket size (Figure S3). The initial coordinates of ligand atoms are sampled from a normal distribution and atom types are sampled from a Gumbel distribution and then transformed into one-hot vectors.

### Featurization of atoms and distance

Atoms in ligand and protein are represented by one-hot vector initially and then transformed by a linear layer. We use a mixed representation for protein atoms and ligand atoms as described in [3]. Specifically, the protein atom features encode the information about amino acid types, atom types (C, N, O, S) and whether the atom is backbone atoms, so the dimension for the input protein atoms are 20+4+1=25 The ligand atom features encode the atom types (C, N, O, F, P, S, Cl) and aromatic information (C, N, O, S, P), and the dimension for ligand atoms is 7+5=12 The distance between atoms and bond types are used to construct graph edges. Four types of edges are considered by one-hot vector, which indicates the connection between ligand atoms, protein atoms, ligand-protein atoms and protein-ligand atoms. The edge feature are then encoded by gaussian radial basis functions with learnable parameters of mean and variance, we use similar structure as described in[4]. Formally, for the distance $d_{ij}$ and edge types $e_{ij}$ are encoded by:

$$\frac{1}{\sqrt{2\pi}\left| \sigma^{k} \right|}exp(-\frac{1}{2}\left( \frac{\alpha_{\left( i,j \right)}d_{ij}+\beta_{\left( i,j \right)}-\mu^{k}}{\left| \sigma^{k} \right|})^{2} \right), k=1,\cdots,K,$$

where $K$ is the number of gaussian radial basis functions, we set $K=15$ in the experiment. $\alpha_{\left( i,j \right)}$ and $\beta_{\left( i,j \right)}$ are learnable scalars indexed by edge types $e_{ij}$, $\mu^{k}$ and $\sigma^{k}$ are the mean and standard variance of $k$th gaussian radial basis function.

### Characterizations and parameters of interactions

In this paper, we consider four types of interactions, and the characterizations of interactions are consistent with BINANA2. Cation-π interactions comprise of a charged functional group and an aromatic ring. The coordinate of charged functional groups is projected to the plane of the aromatic ring and cation-π interaction is accepted if the distance of two center points between pairs is less than a threshold. π-π interactions have two types of forms, pi-pi stacking (face to face) and T-stacking (edge to face). To detect π-π interactions, distance of the projection of center points on two aromatic rings and the angle of two vectors normal to planes for each ring are calculated. If the distance and angle satisfy certain thresholds, a π-π interaction is identified. Hydrogen bond is composed of a hydrogen bond donor and a hydrogen bond acceptor. In BINANA2, thiol, amine, and hydroxyl groups are allowed as donors and nitrogen, sulfur and oxygen atoms can act as receptors. Likewise, the distance between donor and receptor and the dihedral angle between hydrogen atoms, donor and receptor must locate in a certain range. Halogen bonds also consist of a donor and a receptor. The donors include O-X, N-X, S-X, and C-X, where X is F, Cl, Br, or I. The acceptors could be nitrogen, sulfur and oxygen atoms. The threshold of distance for halogen bonds tends to be longer than hydrogen bonds and the dihedral angle is the same. The details of threshold values are listed in Table S2.

## Proof of SE(3)-equivariance of cross attention module in generative Markov transition

The proof of SE(3)-equivariance in node and coordinate updating modules are the same as Guan’s work[5], here we prove the equivariance of cross attention. Denoting the SE(3)-transformation as $T_{g}$, the equivariance of a parametrized module states as $m_{\theta}\left( T_{g}\left( \mathcal{M} \right) \right)={T_{g}(m}_{\theta}\left( \mathcal{M} \right))$ for a protein-ligand complex $\mathcal{M}$. Since the atom features are always SE(3)-invariant, we prove the atom coordinates update in cross attention module.

First, recall that the distance between atoms is encoded by $\text{dis\_encoding}\text{(∙)}$, which is invariant to SE(3) transformation:

$$\tilde{d}_{ij}=\text{dis}\text{\_encoding}\left( ||T_{g}\left( x_{i} \right)-T_{g}\left( x_{j} \right)||^{2} \right)$$

$$=\text{dis}\text{\_encoding}\left( ||(Rx_{i}+b)-(Rx_{j}+b)||^{2} \right)$$

$$=\text{dis}\text{\_encoding}\left( ||Rx_{i}-Rx_{j}||^{2} \right)$$

$$=\text{dis}\text{\_encoding}\text{(}\left( x_{i}-x_{j})^{T}R^{T}R\left( x_{i}-x_{j} \right) \right)$$

$$=\text{dis}\text{\_encoding}\text{(}d_{ij}\text{)},$$

where $R$ is a rotation matrix and $b$ is a translation vector. Similarly, the deviation, $\tilde{x}^{l,\left( L \right)}=mean\left( softmax\left( sim \right)\left[ nheads: \right]\cdot d_{ij} \right)$ is also invariant to SE(3) transformation. Then, for update equation $x^{l,\left( L \right)}=x^{l_{x},\left( L \right)}+[x_{i}^{l_{x},\left( L \right)}-x_{j}^{l_{p},\left( L \right)}]_{ij}\cdot Linear\left( \tilde{x}^{l,\left( L \right)} \right)$, denotes as $\phi_{\theta}(x^{l,\left( L \right)})$, we have:

$$\phi_{\theta}(T_{g}\left( x^{l,\left( L \right)} \right))=T_{g}\left( x^{l-c,\left( L \right)} \right)+[T_{g}(x_{i}^{l_{x},\left( L \right)})-T_{g}(x_{j}^{l_{x},\left( p \right)})]_{ij}\cdot MLP\left( \tilde{x}^{l,\left( L \right)} \right),$$

$$=Rx^{l_{x},\left( L \right)}+b+[Rx_{i}^{l_{x},\left( L \right)}-Rx_{j}^{l_{x},\left( p \right)}]_{ij}\cdot MLP\left( \tilde{x}^{l,\left( L \right)} \right)$$

$$=R(x^{l_{x},\left( L \right)}+[x_{i}^{l_{x},\left( L \right)}-x_{j}^{l_{x},\left( p \right)}]_{ij}\cdot MLP\left( \tilde{x}^{l,\left( L \right)} \right))+b$$

$$=Rx^{l,\left( L \right)}+b$$

$$=T_{g}\left( \phi_{\theta}\left( x^{l,\left( L \right)} \right) \right)$$

So the cross attention is SE(3)-equivariant with respect to the input. By leveraging the conclusion from [6, 7] that a SE(3)-invariant initial density and a SE(3)-equivariant Markov transition function can guarantee an invariant likelihood with respect to $T_{g}$, we can draw a conclusion that our model is likelihood invariant to rotating and translating of protein-ligand complex.

## Reference

1. Vignac, C., et al., *Midi: Mixed graph and 3d denoising diffusion for molecule generation.* 2023.

2. Chen, X., et al., *Symbolic discovery of optimization algorithms.* arXiv preprint arXiv:2302.06675, 2023.

3. Guan, J., et al., *3d equivariant diffusion for target-aware molecule generation and affinity prediction.* arXiv preprint arXiv:2303.03543, 2023.

4. Luo, S., et al., *One transformer can understand both 2d & 3d molecular data.* arXiv preprint arXiv:2210.01765, 2022.

5. Guan, J., et al., *3d equivariant diffusion for target-aware molecule generation and affinity prediction.* 2023.

6. Köhler, J., L. Klein, and F.J.U.h.a.o.a. Noé, *Equivariant flows: Exact likelihood generative learning for symmetric densities, 2020.* 2006.

7. Xu, M., et al., *Geodiff: A geometric diffusion model for molecular conformation generation.* 2022.
